# Crystal structure of *trans*-diammine(1,4,8,11-tetra­aza­cyclo­tetra­decane-κ^4^ *N*)chromium(III) tetra­chlorido­zincate chloride monohydrate from synchrotron data

**DOI:** 10.1107/S205698901600356X

**Published:** 2016-03-04

**Authors:** Dohyun Moon, Jong-Ha Choi

**Affiliations:** a Pohang Accelerator Laboratory, POSTECH, Pohang 37673, Republic of Korea; b Department of Chemistry, Andong National University, Andong 36729, Republic of Korea

**Keywords:** crystal structure, cyclam, ammine ligand, tetra­chlorido­zincate chloride double salt, *trans*-III conformation, chromium(III) complex

## Abstract

The asymmetric unit of the title complex salt, [Cr(C_10_H_24_N_4_)(NH_3_)_2_][ZnCl_4_]Cl·H_2_O, is comprised of four halves of the Cr^III^ complex cations (the counterparts being generated by application of inversion symmetry), two tetra­chlorido­zincate anions, two chloride anions and two water mol­ecules. Each Cr^III^ ion is coordinated by the four N atoms of the cyclam (1,4,8,11-tetra­aza­cyclo­tetra­deca­ne) ligand in the equatorial plane and by two N atoms of ammine ligands in axial positions, displaying an overall distorted octa­hedral coordination environment. The Cr—N(cyclam) bond lengths range from 2.0501 (15) to 2.0615 (15) Å, while the Cr—(NH_3_) bond lengths range from 2.0976 (13) to 2.1062 (13) Å. The macrocyclic cyclam moieties adopt the *trans*-III conformation with six- and five-membered chelate rings in chair and *gauche* conformations. The [ZnCl_4_]^2−^ anions have a slightly distorted tetra­hedral shape. In the crystal, the Cl^−^ anions link the complex cations, as well as the solvent water mol­ecules, through N—H⋯Cl and O—H⋯Cl hydrogen-bonding inter­actions. The supra­molecular set-up also includes N—H⋯Cl, C—H⋯Cl, N—H⋯O and O—H⋯Cl hydrogen bonding between N—H or C—H groups of cyclam, ammine N—H and water O—H donor groups, and O atoms of the water mol­ecules, Cl^−^ anions or Cl atoms of the [ZnCl_4_]^2−^ anions as acceptors, leading to a three-dimensional network structure.

## Abstract 2

Four independent Cr^III^ ions are present in the title structure, each situated on an inversion centre and with a distorted octa­hedral coordination sphere by six N atoms (four from a cyclam ligand in the equatorial plane and two in axial positions). The crystal packing is stabilized by extensive hydrogen-bonding inter­actions between the mol­ecular and ionic moieties.

## Chemical context   

The cyclam macrocycle (1,4,8,11-tetra­aza­cyclo­tetra­decane, C_10_H_24_N_4_) can adopt both planar (*trans*) and folded (*cis*) configurations (Poon & Pun, 1980 ▸). There are five conformational *trans* isomers for the macrocycle, which differ in the chirality of the *sec*-NH groups (Choi, 2009 ▸). The *trans*-I, *trans*-II and *trans*-V conformations can fold to form *cis*-I, *cis*-II and *cis*-V isomers, respectively (Subhan *et al.*, 2011 ▸). Recently, it has been reported that cyclam derivatives and their metal complexes exhibit *anti*-HIV activity (Ronconi & Sadler, 2007 ▸; De Clercq, 2010 ▸; Ross *et al.*, 2012 ▸) whereby the strength of binding to the CXCR4 receptor correlates with the anti-HIV activity. The conformation of the macrocyclic ligand and the orientations of the N—H bonds are very important factors for co-receptor recognition. Therefore, a deeper knowledge of the conformation and crystal packing of metal complexes containing the cyclam ligand has become important in the development of new highly effective anti*-*HIV drugs that specifially target alternative events in the HIV replicative cycle (De Clercq, 2010 ▸). In addition, counter-anionic species play an important role in chemistry, pharmacy and biology (Flores-Velez *et al.*, 1991 ▸; Fabbrizzi & Poggi, 2013 ▸). As part of a study on the structural and supra­molecular features of chromium(III) complex cations with a macrocyclic ligand and with different anions, we report here the structural characterization of *trans-*[Cr(NH_3_)_2_(cyclam)][ZnCl_4_]Cl·H_2_O, (I).
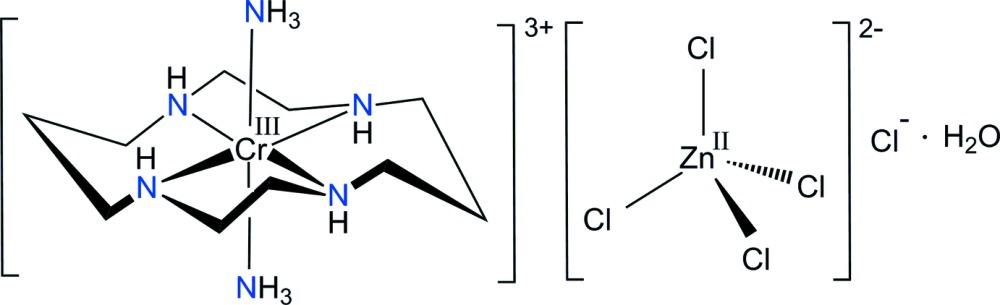


## Structural commentary   

**Figure 1:**
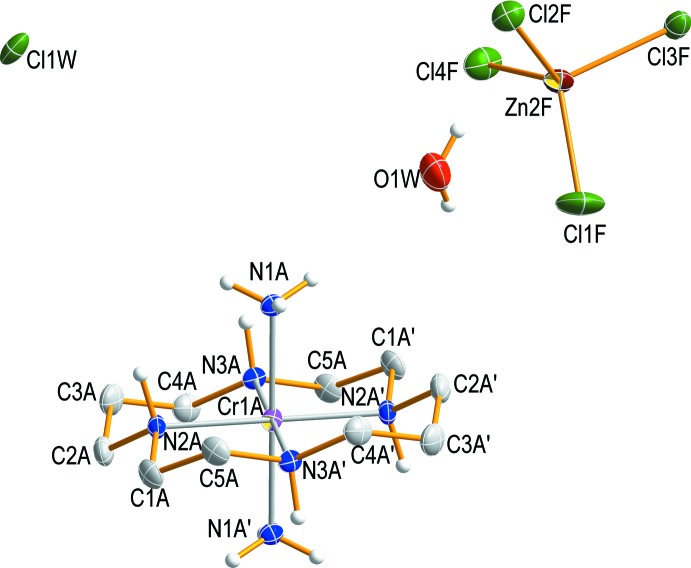
The mol­ecular structures (drawn with displacement ellipsoids at the 50% probability level) of one independent chromium(III) complex cation, one tetra­chlorido­zincate anion, one chloride anion and one water mol­ecule in compound (I). The primed atoms are related by symmetry code (−*x*, −*y* + 1, −*z* + 2).

Compound (I) is another example containing a *trans*-[Cr(NH_3_)_2_(cyclam)]^3+^ moiety but with a different double counter-anion (Derwahl *et al.*, 1999 ▸). The asymmetric unit of (I) comprises four halves of the Cr^III^ complex cations, two tetra­chlorido­zincate anions, two chloride anions and two water mol­ecules. The four Cr atoms are located on crystallographic centers of symmetry. Since the complex cations have mol­ecular *C_i_* symmetry, the cyclam ligand has a *trans*-III conformation (Fig. 1 ▸). In each of the complex cations, the Cr^III^ ion is coordinated by the nitro­gen atoms of the cyclam ligand occupying the equatorial sites. Two ammine ligands complete the distorted *trans*-configured octa­hedral coordination sphere at the axial positions. The Cr—N bond lengths including the donor atoms of the cyclam ligand range from 2.0501 (15) to 2.0615 (15) Å, comparable to those determined for *trans-*[CrCl_2_(cyclam)]_2_[ZnCl_4_] (Flores-Velez *et al.*, 1991 ▸), *trans*-[Cr(nic-O)_2_(cyclam)]ClO_4_ (nic-O = O-coordinating nicotinate; Choi, 2009 ▸), *trans-*[CrF_2_(2,2,3-tet)]ClO_4_ (2,2,3-tet = 1,4,7,11-tetra­aza­undecane; Choi & Moon, 2014 ▸), [Cr(ox)(cyclam)]ClO_4_ (ox = oxalate; Choi *et al.*, 2004 ▸) or [Cr(acac)(cyclam)](ClO_4_)_2_·0.5H_2_O (acac = acetyl­acetonate; Subhan *et al.*, 2011 ▸). However, the Cr—N bond lengths of the secondary amine group of the cyclam ligands are slightly shorter than those of the primary amine group as determined for *trans*-[CrCl_2_(Me_2_tn)_2_]_2_ZnCl_4_ (Me_2_tn = 2,2-di­methyl­propane-1,3-di­amine; Choi *et al.*, 2011 ▸), *trans*-[Cr(N_3_)_2_(Me_2_tn)_2_]ClO_4_·2H_2_O (Moon & Choi, 2015 ▸), or *trans*-[Cr(NCS)_2_(Me_2_tn)_2_]SCN·0.5H_2_O (Choi & Lee, 2009 ▸). The Cr—(NH_3_) bond lengths range from 2.0976 (13) to 2.1062 (13) Å, similar to the average value of 2.095 (3) Å found in *trans*-[Cr(NH_3_)_2_(cyclam)](ClO_4_)Cl_2_ (Derwahl *et al.*, 1999 ▸). The five-membered chelate rings of the cyclam ligands adopt *gauche* and six-membered ring chair conformations. The tetra­hedral [ZnCl_4_]^2−^ anion is distorted due to its involvement in hydrogen-bonding inter­actions. It exhibits Zn—Cl bond lengths ranging from 2.2238 (10) to 2.3232 (8) Å and Cl—Zn—Cl angles from 105.67 (3) to 115.38 (3)°.

## Supra­molecular features   

**Figure 2:**
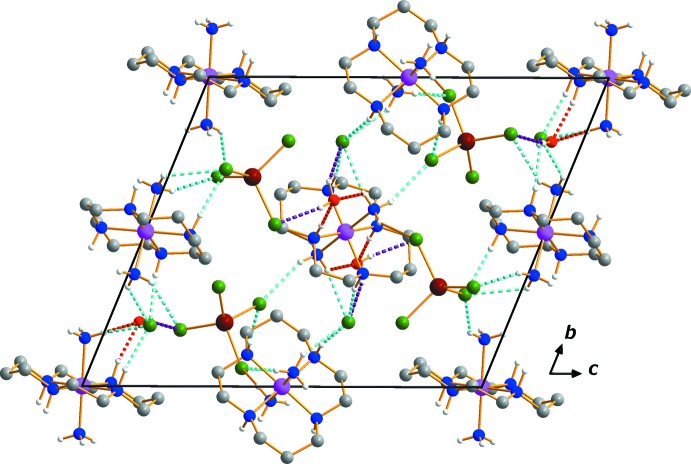
The crystal packing in compound (I), viewed perpendicular to the *bc* plane. Dashed lines represent hydrogen bonding inter­actions N—H⋯Cl (cyan), N—H⋯O (red) and O—H⋯Cl (purple), respectively. H atoms on C atoms have been omitted.

In the crystal, the complex cations are stacked parallel to the *a-*axis direction. A series of N—H⋯Cl and C—H⋯Cl hydrogen bonds link the cations to neighboring anions. An extensive array of additional N—H⋯O and O—H⋯Cl contacts including the lattice water mol­ecule generates a three-dimensional network (Table 1 ▸, Fig. 2 ▸).

## Database survey   

A search in the Cambridge Structural Database (Version 5.36, last update May 2015; Groom & Allen, 2014 ▸) gave just one hit for a [Cr(NH_3_)_2_(cyclam)]^3+^ unit, *viz*. the crystal structure of *trans*-[Cr(NH_3_)_2_(cyclam)](ClO_4_)Cl_2_ (Derwahl *et al.*, 1999 ▸). This dichloride perchlorate double salt and the title compound show the same *trans*-III conformation of the cyclam ligand, however with different hydrogen-bonding and crystal packing networks. The crystal structure of *cis*-[Cr(NH_3_)_2_(cyclam)]I_3_·H_2_O was also found (Kukina *et al.*, 1991 ▸), but no structure of any double salt of *trans*-[Cr(NH_3_)_2_(cyclam)]^3+^ with an additional [ZnCl_4_]^2−^ anion.

## Synthesis and crystallization   

Cyclam and CrCl_3_(THF)_3_ were purchased from Stream Chemicals and used as provided. All chemicals were reagent grade materials and used without further purification. The starting material, *trans-*[Cr(NH_3_)_2_(cyclam)](PF_6_)(NO_3_)·0.5H_2_O, was prepared according to a previously described procedure (Kane-Maguire *et al.*, 1985 ▸). The hexa­fluorido­phosphate nitrate double salt (0.042 g) was dissolved in 5 ml of 0.01 *M* HCl and added to 2 ml of 1 *M* HCl containing 0.12 g of solid ZnCl_2_. The resulting solution was filtered and allowed to stand at room temperature for five days to give block-like yellow crystals of (I) suitable for X-ray structural analysis.

## Refinement   

Crystal data, data collection and structure refinement details are summarized in Table 2 ▸. All H atoms were placed in geometrically idealized positions and constrained to ride on their parent atoms, with C—H = 0.98 Å and N—H = 0.90–0.99 Å and with *U*_iso_(H) values of 1.2 or 1.5 *U*_eq_ of the parent atoms. The hydrogen atoms of water mol­ecules were located in difference maps and restrained with O—H = 0.84 Å using DFIX and DANG commands (Sheldrick, 2015*b* ▸).

## Supplementary Material

Crystal structure: contains datablock(s) I. DOI: 10.1107/S205698901600356X/wm5272sup1.cif

Structure factors: contains datablock(s) I. DOI: 10.1107/S205698901600356X/wm5272Isup2.hkl

CCDC reference: 1456673

Additional supporting information:  crystallographic information; 3D view; checkCIF report

## Figures and Tables

**Table 1:** Hydrogen-bond geometry (Å, °)

*D*—H⋯*A*	*D*—H	H⋯*A*	*D*⋯*A*	*D*—H⋯*A*
N1*A*—H1*NA*⋯Cl3*F* ^i^	0.90	2.80	3.3602 (16)	121
N1*A*—H1*NA*⋯Cl2*W* ^ii^	0.90	2.60	3.3363 (18)	140
N1*A*—H3*NA*⋯Cl3*E* ^iii^	0.90	2.58	3.384 (2)	149
N2*A*—H1*A*⋯Cl2*W* ^ii^	0.99	2.20	3.1754 (15)	170
N3*A*—H2*A*⋯Cl3*F* ^i^	0.99	2.30	3.2752 (14)	170
C1*A*—H1*A*2⋯Cl4*E* ^ii^	0.98	2.59	3.4739 (19)	150
N1*B*—H2*NB*⋯Cl1*W* ^iv^	0.90	2.45	3.2683 (19)	152
N1*B*—H2*NB*⋯O1*W*	0.90	2.46	3.034 (2)	122
N1*B*—H3*NB*⋯Cl2*E* ^iv^	0.90	2.57	3.3556 (15)	147
N2*B*—H1*B*⋯Cl1*W*	0.99	2.20	3.1704 (17)	165
N3*B*—H2*B*⋯O1*W* ^iv^	0.99	1.98	2.968 (2)	177
N1*C*—H2*NC*⋯Cl3*F* ^v^	0.90	2.68	3.4211 (19)	140
N1*C*—H3*NC*⋯Cl2*W* ^vi^	0.90	2.38	3.2673 (17)	167
N1*C*—H3*NC*⋯O2*W* ^vi^	0.90	2.54	2.975 (2)	111
N2*C*—H1*C*⋯O2*W* ^vii^	0.99	1.96	2.932 (2)	167
N3*C*—H2*C*⋯Cl2*W* ^vii^	0.99	2.23	3.2082 (16)	171
C5*C*—H5*C*2⋯Cl4*F*	0.98	2.79	3.761 (2)	174
N1*D*—H2*ND*⋯Cl1*W* ^viii^	0.90	2.45	3.2796 (15)	154
N1*D*—H3*ND*⋯Cl1*E* ^viii^	0.90	2.71	3.5516 (16)	156
N2*D*—H1*D*⋯Cl1*W* ^ix^	0.99	2.19	3.1589 (16)	166
N3*D*—H2*D*⋯Cl2*E* ^ix^	0.99	2.36	3.3276 (17)	166
C1*D*—H1*D*1⋯Cl1*F* ^i^	0.98	2.66	3.6278 (17)	168
C1*D*—H1*D*2⋯Cl1*E* ^viii^	0.98	2.83	3.803 (2)	172
O1*W*—H1*O*1⋯Cl1*W* ^iv^	0.85 (1)	2.70 (2)	3.341 (2)	134 (2)
O1*W*—H2*O*1⋯Cl2*F*	0.84 (1)	2.39 (1)	3.2066 (17)	167 (2)
O2*W*—H1*O*2⋯Cl3*E*	0.83 (1)	2.37 (1)	3.1763 (19)	162 (2)
O2*W*—H2*O*2⋯Cl2*W*	0.84 (1)	2.55 (2)	3.2009 (19)	135 (2)

Symmetry codes: (i) 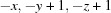; (ii) 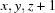; (iii) 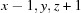; (iv) 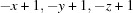; (v) 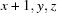; (vi) 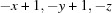; (vii) 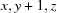; (viii) 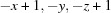; (ix) 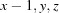.

**Table 2:** Experimental details

Crystal data	
Chemical formula	[Cr(C_10_H_24_N_4_)(NH_3_)_2_][ZnCl_4_]Cl·H_2_O
*M*_r_	547.03
Crystal system, space group	Triclinic, *P* 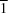
Temperature (K)	243
*a*, *b*, *c* (Å)	9.3980 (19), 14.876 (3), 17.981 (4)
α, β, γ (°)	66.03 (3), 76.03 (3), 78.74 (3)
*V* (Å^3^)	2215.6 (10)
*Z*	4
Radiation type	Synchrotron, λ = 0.620 Å
μ (mm^−1^)	1.50
Crystal size (mm)	0.11 × 0.08 × 0.04

Data collection	
Diffractometer	ADSC Q210 CCD area-detector
Absorption correction	Empirical (using intensity measurements) (*HKL3000sm *SCALEPACK**; Otwinowski & Minor, 1997 ▸)
*T*_min_, *T*_max_	0.850, 0.938
No. of measured, independent and observed [*I* > 2σ(*I*)] reflections	23883, 12905, 11123
*R*_int_	0.029
(sin θ/λ)_max_ (Å^−1^)	0.707

Refinement	
*R*[*F* ^2^ > 2σ(*F* ^2^)], *wR*(*F* ^2^), *S*	0.029, 0.084, 1.07
No. of reflections	12905
No. of parameters	455
No. of restraints	6
H-atom treatment	H atoms treated by a mixture of independent and constrained refinement
Δρ_max_, Δρ_min_ (e Å^−3^)	0.66, −0.76

Computer programs: *PAL BL2D-SMDC Program* (Shin *et al.*, 2016 ▸), *HKL3000sm* (Otwinowski & Minor, 1997 ▸), *SHELXT2014* (Sheldrick, 2015*a* ▸), *SHELXL2014* (Sheldrick, 2015*b* ▸), *DIAMOND* (Putz & Brandenburg, 2014 ▸) and *publCIF* (Westrip, 2010 ▸).

## supplementary crystallographic information

### Crystal data

**Table:**

[Cr(C_10_H_24_N_4_)(NH_3_)_2_][ZnCl_4_]Cl·H_2_O	*Z* = 4
*M**_r_* = 547	*F*(000) = 1124
Triclinic, *P*1	*D*_x_ = 1.640 Mg m^−^^3^
*a* = 9.3980 (19) Å	Synchrotron radiation, λ = 0.620 Å
*b* = 14.876 (3) Å	Cell parameters from 76389 reflections
*c* = 17.981 (4) Å	θ = 0.4–33.6°
α = 66.03 (3)°	µ = 1.50 mm^−^^1^
β = 76.03 (3)°	*T* = 243 K
γ = 78.74 (3)°	Block, yellow
*V* = 2215.6 (10) Å^3^	0.11 × 0.08 × 0.04 mm

### Data collection

**Table:**

ADSC Q210 CCD area-detector diffractometer	11123 reflections with *I* > 2σ(*I*)
Radiation source: PLSII 2D bending magnet	*R*_int_ = 0.029
ω scan	θ_max_ = 26.0°, θ_min_ = 1.1°
Absorption correction: empirical (using intensity measurements) (*HKL3000sm *SCALEPACK**; Otwinowski & Minor, 1997)	*h* = −13→13
*T*_min_ = 0.850, *T*_max_ = 0.938	*k* = −21→21
23883 measured reflections	*l* = −25→25
12905 independent reflections	

### Refinement

**Table:**

Refinement on *F*^2^	6 restraints
Least-squares matrix: full	Hydrogen site location: mixed
*R*[*F*^2^ > 2σ(*F*^2^)] = 0.029	H atoms treated by a mixture of independent and constrained refinement
*wR*(*F*^2^) = 0.084	*w* = 1/[σ^2^(*F*_o_^2^) + (0.0513*P*)^2^ + 0.1347*P*] where *P* = (*F*_o_^2^ + 2*F*_c_^2^)/3
*S* = 1.07	(Δ/σ)_max_ = 0.002
12905 reflections	Δρ_max_ = 0.66 e Å^−^^3^
455 parameters	Δρ_min_ = −0.76 e Å^−^^3^

### Special details

**Table:**

Geometry. All e.s.d.'s (except the e.s.d. in the dihedral angle between two l.s. planes) are estimated using the full covariance matrix. The cell e.s.d.'s are taken into account individually in the estimation of e.s.d.'s in distances, angles and torsion angles; correlations between e.s.d.'s in cell parameters are only used when they are defined by crystal symmetry. An approximate (isotropic) treatment of cell e.s.d.'s is used for estimating e.s.d.'s involving l.s. planes.

### Fractional atomic coordinates and isotropic or equivalent isotropic displacement parameters (Å^2^)

**Table:**

	*x*	*y*	*z*	*U*_iso_*/*U*_eq_	
Cr1A	0.0000	0.5000	1.0000	0.00997 (6)	
N1A	−0.05398 (13)	0.36162 (9)	1.01590 (7)	0.0164 (2)	
H1NA	0.0293	0.3205	1.0125	0.025*	
H2NA	−0.1031	0.3695	0.9761	0.025*	
H3NA	−0.1111	0.3358	1.0658	0.025*	
N2A	0.17264 (13)	0.42870 (9)	1.06157 (7)	0.0167 (2)	
H1A	0.1790	0.3582	1.0695	0.020*	
N3A	0.12143 (13)	0.51146 (9)	0.88534 (7)	0.0157 (2)	
H2A	0.1219	0.4474	0.8806	0.019*	
C1A	0.12606 (17)	0.43212 (12)	1.14593 (9)	0.0227 (3)	
H1A1	0.1385	0.4971	1.1438	0.027*	
H1A2	0.1870	0.3815	1.1838	0.027*	
C2A	0.32146 (16)	0.46018 (12)	1.01888 (10)	0.0238 (3)	
H2A1	0.3946	0.4181	1.0528	0.029*	
H2A2	0.3230	0.5286	1.0127	0.029*	
C3A	0.36314 (16)	0.45343 (13)	0.93376 (11)	0.0273 (3)	
H3A1	0.4686	0.4605	0.9136	0.033*	
H3A2	0.3483	0.3871	0.9400	0.033*	
C4A	0.27855 (16)	0.52922 (12)	0.86811 (9)	0.0238 (3)	
H4A1	0.2832	0.5956	0.8656	0.029*	
H4A2	0.3257	0.5267	0.8140	0.029*	
C5A	0.03428 (17)	0.58654 (12)	0.82368 (8)	0.0218 (3)	
H5A1	0.0693	0.5810	0.7696	0.026*	
H5A2	0.0457	0.6533	0.8177	0.026*	
Cr2B	0.5000	0.5000	0.5000	0.01185 (6)	
N1B	0.30918 (13)	0.57334 (9)	0.54783 (7)	0.0188 (2)	
H1NB	0.2410	0.5307	0.5761	0.028*	
H2NB	0.2731	0.6239	0.5060	0.028*	
H3NB	0.3315	0.5969	0.5820	0.028*	
N2B	0.44033 (14)	0.36582 (9)	0.58615 (8)	0.0204 (2)	
H1B	0.5253	0.3162	0.5812	0.024*	
N3B	0.62099 (14)	0.51975 (10)	0.57270 (8)	0.0218 (2)	
H2B	0.7178	0.4803	0.5666	0.026*	
C1B	0.3178 (2)	0.34242 (13)	0.55956 (12)	0.0317 (4)	
H1B1	0.2242	0.3773	0.5768	0.038*	
H1B2	0.3095	0.2712	0.5856	0.038*	
C2B	0.40555 (19)	0.35503 (13)	0.67422 (10)	0.0299 (4)	
H2B1	0.3851	0.2869	0.7092	0.036*	
H2B2	0.3163	0.3990	0.6828	0.036*	
C3B	0.5303 (2)	0.37936 (14)	0.70072 (10)	0.0318 (4)	
H3B1	0.6206	0.3389	0.6873	0.038*	
H3B2	0.5079	0.3591	0.7610	0.038*	
C4B	0.5622 (2)	0.48701 (14)	0.66301 (10)	0.0300 (4)	
H4B1	0.4712	0.5291	0.6725	0.036*	
H4B2	0.6341	0.4950	0.6907	0.036*	
C5B	0.6501 (2)	0.62542 (13)	0.53436 (12)	0.0315 (4)	
H5B1	0.7347	0.6335	0.5531	0.038*	
H5B2	0.5640	0.6667	0.5511	0.038*	
Cr3C	0.5000	1.0000	0.0000	0.01576 (6)	
N1C	0.54753 (15)	0.84509 (9)	0.04233 (8)	0.0240 (3)	
H1NC	0.4628	0.8170	0.0595	0.036*	
H2NC	0.5966	0.8240	0.0847	0.036*	
H3NC	0.6036	0.8280	0.0009	0.036*	
N2C	0.63796 (15)	1.01044 (11)	0.06889 (8)	0.0256 (3)	
H1C	0.6218	1.0801	0.0645	0.031*	
N3C	0.31688 (15)	0.98526 (10)	0.09225 (8)	0.0243 (3)	
H2C	0.2794	1.0527	0.0913	0.029*	
C1C	0.79153 (19)	0.99551 (16)	0.02512 (12)	0.0362 (4)	
H1C1	0.8224	0.9246	0.0388	0.043*	
H1C2	0.8590	1.0222	0.0429	0.043*	
C2C	0.6153 (2)	0.94799 (15)	0.15942 (11)	0.0342 (4)	
H2C1	0.6799	0.9657	0.1856	0.041*	
H2C2	0.6432	0.8783	0.1669	0.041*	
C3C	0.4561 (2)	0.96092 (15)	0.20207 (10)	0.0352 (4)	
H3C1	0.4520	0.9271	0.2619	0.042*	
H3C2	0.4278	1.0316	0.1908	0.042*	
C4C	0.3413 (2)	0.92345 (13)	0.17816 (10)	0.0323 (4)	
H4C1	0.3738	0.8550	0.1827	0.039*	
H4C2	0.2479	0.9238	0.2168	0.039*	
C5C	0.20231 (19)	0.95212 (15)	0.06752 (12)	0.0338 (4)	
H5C1	0.1045	0.9676	0.0970	0.041*	
H5C2	0.2198	0.8803	0.0822	0.041*	
Cr4D	0.0000	0.0000	0.5000	0.00919 (6)	
N1D	0.22787 (12)	−0.04570 (9)	0.48598 (7)	0.0160 (2)	
H1ND	0.2753	−0.0002	0.4410	0.024*	
H2ND	0.2450	−0.1042	0.4798	0.024*	
H3ND	0.2609	−0.0523	0.5311	0.024*	
N2D	0.02941 (13)	0.09509 (9)	0.55052 (8)	0.0177 (2)	
H1D	−0.0667	0.1356	0.5558	0.021*	
N3D	0.02438 (12)	0.10266 (9)	0.38037 (7)	0.0155 (2)	
H2D	−0.0727	0.1423	0.3738	0.019*	
C1D	0.05317 (18)	0.03371 (13)	0.63680 (10)	0.0255 (3)	
H1D1	0.0337	0.0752	0.6695	0.031*	
H1D2	0.1555	0.0031	0.6363	0.031*	
C2D	0.13974 (19)	0.16666 (12)	0.50160 (11)	0.0284 (3)	
H2D1	0.2385	0.1305	0.4978	0.034*	
H2D2	0.1382	0.2108	0.5301	0.034*	
C3D	0.1080 (2)	0.22836 (12)	0.41438 (12)	0.0320 (4)	
H3D1	0.0048	0.2573	0.4193	0.038*	
H3D2	0.1694	0.2832	0.3893	0.038*	
C4D	0.13418 (17)	0.17441 (12)	0.35548 (10)	0.0264 (3)	
H4D1	0.1281	0.2229	0.2994	0.032*	
H4D2	0.2337	0.1390	0.3545	0.032*	
C5D	0.05122 (17)	0.04541 (12)	0.32576 (9)	0.0238 (3)	
H5D1	0.1536	0.0149	0.3213	0.029*	
H5D2	0.0340	0.0896	0.2702	0.029*	
Zn1E	0.61151 (2)	0.20650 (2)	0.29082 (2)	0.02403 (5)	
Cl1E	0.53804 (5)	0.05903 (4)	0.38072 (3)	0.04285 (12)	
Cl2E	0.72540 (4)	0.26657 (3)	0.35901 (2)	0.02630 (8)	
Cl3E	0.79024 (5)	0.18205 (4)	0.18756 (3)	0.03439 (9)	
Cl4E	0.43263 (5)	0.32125 (4)	0.23927 (3)	0.04760 (14)	
Zn2F	0.01806 (2)	0.67654 (2)	0.21094 (2)	0.02221 (5)	
Cl1F	−0.02844 (7)	0.79302 (4)	0.26421 (3)	0.04689 (13)	
Cl2F	0.00187 (5)	0.52354 (3)	0.31592 (2)	0.02954 (8)	
Cl3F	−0.16681 (4)	0.70070 (3)	0.13702 (2)	0.02651 (8)	
Cl4F	0.23650 (5)	0.67718 (4)	0.12345 (3)	0.03785 (10)	
Cl1W	0.72406 (5)	0.20867 (3)	0.60004 (2)	0.02949 (9)	
Cl2W	0.20883 (5)	0.19663 (3)	0.10707 (3)	0.03019 (9)	
O1W	0.09042 (18)	0.60251 (13)	0.43830 (10)	0.0463 (4)	
H1O1	0.103 (3)	0.6621 (9)	0.4071 (13)	0.056*	
H2O1	0.055 (3)	0.5784 (17)	0.4128 (14)	0.056*	
O2W	0.55603 (19)	0.20704 (13)	0.07745 (10)	0.0473 (4)	
H1O2	0.6248 (18)	0.211 (2)	0.0971 (15)	0.057*	
H2O2	0.4729 (15)	0.215 (2)	0.1061 (14)	0.057*	

### Atomic displacement parameters (Å^2^)

**Table:**

	*U*^11^	*U*^22^	*U*^33^	*U*^12^	*U*^13^	*U*^23^
Cr1A	0.00887 (12)	0.00962 (12)	0.00992 (12)	−0.00051 (9)	−0.00151 (9)	−0.00252 (10)
N1A	0.0176 (5)	0.0146 (5)	0.0162 (5)	−0.0023 (4)	−0.0015 (4)	−0.0056 (4)
N2A	0.0132 (5)	0.0156 (5)	0.0202 (5)	0.0000 (4)	−0.0071 (4)	−0.0043 (5)
N3A	0.0147 (5)	0.0159 (5)	0.0150 (5)	−0.0033 (4)	0.0015 (4)	−0.0061 (4)
C1A	0.0261 (7)	0.0253 (7)	0.0168 (6)	−0.0044 (6)	−0.0111 (5)	−0.0031 (6)
C2A	0.0131 (6)	0.0249 (7)	0.0320 (8)	−0.0014 (5)	−0.0076 (5)	−0.0078 (6)
C3A	0.0127 (6)	0.0316 (8)	0.0361 (8)	0.0008 (6)	0.0006 (6)	−0.0155 (7)
C4A	0.0163 (6)	0.0289 (8)	0.0241 (7)	−0.0065 (5)	0.0062 (5)	−0.0117 (6)
C5A	0.0280 (7)	0.0240 (7)	0.0113 (6)	−0.0066 (6)	−0.0044 (5)	−0.0021 (5)
Cr2B	0.01081 (12)	0.01190 (13)	0.01273 (13)	−0.00091 (10)	0.00002 (10)	−0.00589 (11)
N1B	0.0173 (5)	0.0188 (6)	0.0183 (5)	0.0006 (4)	0.0005 (4)	−0.0081 (5)
N2B	0.0189 (6)	0.0160 (6)	0.0209 (6)	−0.0024 (4)	0.0027 (4)	−0.0048 (5)
N3B	0.0206 (6)	0.0259 (6)	0.0243 (6)	−0.0011 (5)	−0.0058 (5)	−0.0147 (5)
C1B	0.0276 (8)	0.0266 (8)	0.0400 (9)	−0.0135 (6)	0.0015 (7)	−0.0115 (7)
C2B	0.0306 (8)	0.0269 (8)	0.0184 (7)	0.0007 (6)	0.0054 (6)	−0.0018 (6)
C3B	0.0370 (9)	0.0351 (9)	0.0174 (7)	0.0065 (7)	−0.0056 (6)	−0.0081 (7)
C4B	0.0353 (9)	0.0363 (9)	0.0230 (7)	0.0062 (7)	−0.0104 (6)	−0.0175 (7)
C5B	0.0338 (9)	0.0286 (8)	0.0420 (10)	−0.0100 (7)	−0.0075 (7)	−0.0199 (8)
Cr3C	0.01463 (14)	0.01256 (14)	0.01522 (14)	0.00175 (11)	−0.00218 (11)	−0.00211 (11)
N1C	0.0272 (6)	0.0172 (6)	0.0221 (6)	0.0031 (5)	−0.0050 (5)	−0.0042 (5)
N2C	0.0237 (6)	0.0261 (7)	0.0250 (6)	−0.0005 (5)	−0.0076 (5)	−0.0069 (6)
N3C	0.0221 (6)	0.0203 (6)	0.0228 (6)	0.0000 (5)	0.0018 (5)	−0.0048 (5)
C1C	0.0201 (7)	0.0428 (11)	0.0411 (10)	0.0004 (7)	−0.0091 (7)	−0.0111 (9)
C2C	0.0407 (10)	0.0337 (9)	0.0252 (8)	0.0004 (8)	−0.0146 (7)	−0.0051 (7)
C3C	0.0482 (11)	0.0321 (9)	0.0191 (7)	−0.0011 (8)	−0.0037 (7)	−0.0061 (7)
C4C	0.0388 (9)	0.0270 (8)	0.0192 (7)	−0.0024 (7)	0.0040 (6)	−0.0024 (6)
C5C	0.0198 (7)	0.0373 (10)	0.0372 (9)	−0.0077 (7)	0.0014 (6)	−0.0087 (8)
Cr4D	0.00721 (11)	0.00831 (12)	0.01150 (12)	−0.00106 (9)	−0.00070 (9)	−0.00365 (10)
N1D	0.0103 (5)	0.0159 (5)	0.0192 (5)	−0.0007 (4)	−0.0014 (4)	−0.0050 (4)
N2D	0.0163 (5)	0.0159 (5)	0.0249 (6)	−0.0011 (4)	−0.0047 (4)	−0.0116 (5)
N3D	0.0123 (5)	0.0140 (5)	0.0156 (5)	−0.0012 (4)	−0.0018 (4)	−0.0015 (4)
C1D	0.0275 (7)	0.0327 (8)	0.0244 (7)	0.0014 (6)	−0.0093 (6)	−0.0183 (7)
C2D	0.0268 (8)	0.0224 (7)	0.0427 (9)	−0.0117 (6)	−0.0071 (7)	−0.0145 (7)
C3D	0.0342 (9)	0.0152 (7)	0.0440 (10)	−0.0122 (6)	−0.0086 (7)	−0.0037 (7)
C4D	0.0209 (7)	0.0220 (7)	0.0255 (7)	−0.0094 (6)	−0.0011 (6)	0.0032 (6)
C5D	0.0231 (7)	0.0316 (8)	0.0140 (6)	0.0018 (6)	−0.0018 (5)	−0.0090 (6)
Zn1E	0.01617 (8)	0.02918 (10)	0.01970 (9)	−0.00577 (7)	−0.00537 (6)	0.00052 (7)
Cl1E	0.02366 (19)	0.0392 (2)	0.0439 (3)	−0.01488 (18)	−0.00507 (17)	0.0108 (2)
Cl2E	0.02669 (18)	0.02502 (18)	0.02753 (18)	0.00389 (14)	−0.01067 (14)	−0.01004 (15)
Cl3E	0.0306 (2)	0.0393 (2)	0.02640 (19)	−0.00741 (17)	0.00146 (15)	−0.00763 (18)
Cl4E	0.02096 (19)	0.0463 (3)	0.0538 (3)	−0.00297 (18)	−0.01940 (19)	0.0098 (2)
Zn2F	0.02487 (9)	0.02355 (9)	0.02018 (9)	−0.00586 (7)	0.00014 (6)	−0.01115 (7)
Cl1F	0.0642 (3)	0.0419 (3)	0.0483 (3)	−0.0180 (2)	0.0050 (2)	−0.0338 (2)
Cl2F	0.0346 (2)	0.02632 (19)	0.02337 (17)	−0.00454 (15)	−0.00178 (15)	−0.00646 (15)
Cl3F	0.02952 (18)	0.02757 (18)	0.02467 (17)	0.00596 (14)	−0.00829 (14)	−0.01429 (15)
Cl4F	0.0297 (2)	0.0382 (2)	0.0430 (2)	−0.01085 (17)	0.01168 (17)	−0.0196 (2)
Cl1W	0.0332 (2)	0.02203 (17)	0.02665 (18)	0.01129 (15)	−0.00334 (15)	−0.01016 (15)
Cl2W	0.0311 (2)	0.01957 (17)	0.0317 (2)	0.00488 (14)	−0.00070 (15)	−0.00768 (15)
O1W	0.0420 (8)	0.0565 (10)	0.0491 (9)	0.0026 (7)	−0.0231 (7)	−0.0239 (8)
O2W	0.0468 (9)	0.0571 (10)	0.0496 (9)	−0.0097 (8)	−0.0099 (7)	−0.0294 (8)

### Geometric parameters (Å, º)

**Table:**

Cr1A—N2A^i^	2.0553 (13)	N1C—H1NC	0.9000
Cr1A—N2A	2.0553 (13)	N1C—H2NC	0.9000
Cr1A—N3A^i^	2.0582 (13)	N1C—H3NC	0.9000
Cr1A—N3A	2.0582 (13)	N2C—C1C	1.490 (2)
Cr1A—N1A	2.1062 (13)	N2C—C2C	1.496 (2)
Cr1A—N1A^i^	2.1062 (13)	N2C—H1C	0.9900
N1A—H1NA	0.9000	N3C—C5C	1.489 (2)
N1A—H2NA	0.9000	N3C—C4C	1.490 (2)
N1A—H3NA	0.9000	N3C—H2C	0.9900
N2A—C2A	1.4866 (19)	C1C—C5C^iii^	1.517 (3)
N2A—C1A	1.4928 (19)	C1C—H1C1	0.9800
N2A—H1A	0.9900	C1C—H1C2	0.9800
N3A—C4A	1.4869 (19)	C2C—C3C	1.522 (3)
N3A—C5A	1.491 (2)	C2C—H2C1	0.9800
N3A—H2A	0.9900	C2C—H2C2	0.9800
C1A—C5A^i^	1.513 (2)	C3C—C4C	1.521 (3)
C1A—H1A1	0.9800	C3C—H3C1	0.9800
C1A—H1A2	0.9800	C3C—H3C2	0.9800
C2A—C3A	1.525 (2)	C4C—H4C1	0.9800
C2A—H2A1	0.9800	C4C—H4C2	0.9800
C2A—H2A2	0.9800	C5C—C1C^iii^	1.517 (3)
C3A—C4A	1.523 (2)	C5C—H5C1	0.9800
C3A—H3A1	0.9800	C5C—H5C2	0.9800
C3A—H3A2	0.9800	Cr4D—N2D^iv^	2.0544 (12)
C4A—H4A1	0.9800	Cr4D—N2D	2.0544 (12)
C4A—H4A2	0.9800	Cr4D—N3D	2.0593 (14)
C5A—C1A^i^	1.513 (2)	Cr4D—N3D^iv^	2.0593 (14)
C5A—H5A1	0.9800	Cr4D—N1D^iv^	2.1029 (12)
C5A—H5A2	0.9800	Cr4D—N1D	2.1029 (12)
Cr2B—N2B^ii^	2.0501 (15)	N1D—H1ND	0.9000
Cr2B—N2B	2.0502 (15)	N1D—H2ND	0.9000
Cr2B—N3B	2.0611 (13)	N1D—H3ND	0.9000
Cr2B—N3B^ii^	2.0611 (13)	N2D—C2D	1.487 (2)
Cr2B—N1B	2.0976 (13)	N2D—C1D	1.492 (2)
Cr2B—N1B^ii^	2.0977 (13)	N2D—H1D	0.9900
N1B—H1NB	0.9000	N3D—C4D	1.491 (2)
N1B—H2NB	0.9000	N3D—C5D	1.4932 (19)
N1B—H3NB	0.9000	N3D—H2D	0.9900
N2B—C2B	1.485 (2)	C1D—C5D^iv^	1.513 (2)
N2B—C1B	1.494 (2)	C1D—H1D1	0.9800
N2B—H1B	0.9900	C1D—H1D2	0.9800
N3B—C4B	1.486 (2)	C2D—C3D	1.530 (3)
N3B—C5B	1.489 (2)	C2D—H2D1	0.9800
N3B—H2B	0.9900	C2D—H2D2	0.9800
C1B—C5B^ii^	1.525 (3)	C3D—C4D	1.521 (3)
C1B—H1B1	0.9800	C3D—H3D1	0.9800
C1B—H1B2	0.9800	C3D—H3D2	0.9800
C2B—C3B	1.519 (3)	C4D—H4D1	0.9800
C2B—H2B1	0.9800	C4D—H4D2	0.9800
C2B—H2B2	0.9800	C5D—C1D^iv^	1.513 (2)
C3B—C4B	1.524 (3)	C5D—H5D1	0.9800
C3B—H3B1	0.9800	C5D—H5D2	0.9800
C3B—H3B2	0.9800	Zn1E—Cl4E	2.2238 (10)
C4B—H4B1	0.9800	Zn1E—Cl1E	2.2523 (11)
C4B—H4B2	0.9800	Zn1E—Cl3E	2.2817 (9)
C5B—C1B^ii^	1.525 (3)	Zn1E—Cl2E	2.3118 (7)
C5B—H5B1	0.9800	Zn2F—Cl1F	2.2275 (7)
C5B—H5B2	0.9800	Zn2F—Cl4F	2.2640 (9)
Cr3C—N3C^iii^	2.0579 (15)	Zn2F—Cl2F	2.2960 (11)
Cr3C—N3C	2.0579 (15)	Zn2F—Cl3F	2.3232 (8)
Cr3C—N2C^iii^	2.0615 (15)	O1W—H1O1	0.848 (9)
Cr3C—N2C	2.0615 (15)	O1W—H2O1	0.836 (9)
Cr3C—N1C	2.1039 (14)	O2W—H1O2	0.832 (9)
Cr3C—N1C^iii^	2.1039 (14)	O2W—H2O2	0.843 (9)
			
N2A^i^—Cr1A—N2A	180.0	N3C—Cr3C—N1C	90.06 (6)
N2A^i^—Cr1A—N3A^i^	94.49 (5)	N2C^iii^—Cr3C—N1C	88.08 (6)
N2A—Cr1A—N3A^i^	85.51 (5)	N2C—Cr3C—N1C	91.92 (6)
N2A^i^—Cr1A—N3A	85.51 (5)	N3C^iii^—Cr3C—N1C^iii^	90.05 (6)
N2A—Cr1A—N3A	94.49 (5)	N3C—Cr3C—N1C^iii^	89.94 (6)
N3A^i^—Cr1A—N3A	180.00 (4)	N2C^iii^—Cr3C—N1C^iii^	91.92 (6)
N2A^i^—Cr1A—N1A	90.76 (5)	N2C—Cr3C—N1C^iii^	88.08 (6)
N2A—Cr1A—N1A	89.24 (5)	N1C—Cr3C—N1C^iii^	180.00 (8)
N3A^i^—Cr1A—N1A	91.07 (6)	Cr3C—N1C—H1NC	109.5
N3A—Cr1A—N1A	88.93 (6)	Cr3C—N1C—H2NC	109.5
N2A^i^—Cr1A—N1A^i^	89.24 (5)	H1NC—N1C—H2NC	109.5
N2A—Cr1A—N1A^i^	90.76 (5)	Cr3C—N1C—H3NC	109.5
N3A^i^—Cr1A—N1A^i^	88.93 (6)	H1NC—N1C—H3NC	109.5
N3A—Cr1A—N1A^i^	91.07 (6)	H2NC—N1C—H3NC	109.5
N1A—Cr1A—N1A^i^	180.00 (6)	C1C—N2C—C2C	113.16 (14)
Cr1A—N1A—H1NA	109.5	C1C—N2C—Cr3C	106.52 (11)
Cr1A—N1A—H2NA	109.5	C2C—N2C—Cr3C	117.72 (12)
H1NA—N1A—H2NA	109.5	C1C—N2C—H1C	106.2
Cr1A—N1A—H3NA	109.5	C2C—N2C—H1C	106.2
H1NA—N1A—H3NA	109.5	Cr3C—N2C—H1C	106.2
H2NA—N1A—H3NA	109.5	C5C—N3C—C4C	113.35 (14)
C2A—N2A—C1A	114.29 (12)	C5C—N3C—Cr3C	107.00 (10)
C2A—N2A—Cr1A	117.18 (9)	C4C—N3C—Cr3C	116.36 (11)
C1A—N2A—Cr1A	106.44 (9)	C5C—N3C—H2C	106.5
C2A—N2A—H1A	106.0	C4C—N3C—H2C	106.5
C1A—N2A—H1A	106.0	Cr3C—N3C—H2C	106.5
Cr1A—N2A—H1A	106.0	N2C—C1C—C5C^iii^	109.33 (14)
C4A—N3A—C5A	113.52 (12)	N2C—C1C—H1C1	109.8
C4A—N3A—Cr1A	117.51 (9)	C5C^iii^—C1C—H1C1	109.8
C5A—N3A—Cr1A	106.14 (9)	N2C—C1C—H1C2	109.8
C4A—N3A—H2A	106.3	C5C^iii^—C1C—H1C2	109.8
C5A—N3A—H2A	106.3	H1C1—C1C—H1C2	108.3
Cr1A—N3A—H2A	106.3	N2C—C2C—C3C	112.24 (15)
N2A—C1A—C5A^i^	108.42 (12)	N2C—C2C—H2C1	109.2
N2A—C1A—H1A1	110.0	C3C—C2C—H2C1	109.2
C5A^i^—C1A—H1A1	110.0	N2C—C2C—H2C2	109.2
N2A—C1A—H1A2	110.0	C3C—C2C—H2C2	109.2
C5A^i^—C1A—H1A2	110.0	H2C1—C2C—H2C2	107.9
H1A1—C1A—H1A2	108.4	C4C—C3C—C2C	116.90 (15)
N2A—C2A—C3A	111.60 (13)	C4C—C3C—H3C1	108.1
N2A—C2A—H2A1	109.3	C2C—C3C—H3C1	108.1
C3A—C2A—H2A1	109.3	C4C—C3C—H3C2	108.1
N2A—C2A—H2A2	109.3	C2C—C3C—H3C2	108.1
C3A—C2A—H2A2	109.3	H3C1—C3C—H3C2	107.3
H2A1—C2A—H2A2	108.0	N3C—C4C—C3C	111.92 (15)
C4A—C3A—C2A	115.86 (13)	N3C—C4C—H4C1	109.2
C4A—C3A—H3A1	108.3	C3C—C4C—H4C1	109.2
C2A—C3A—H3A1	108.3	N3C—C4C—H4C2	109.2
C4A—C3A—H3A2	108.3	C3C—C4C—H4C2	109.2
C2A—C3A—H3A2	108.3	H4C1—C4C—H4C2	107.9
H3A1—C3A—H3A2	107.4	N3C—C5C—C1C^iii^	109.26 (15)
N3A—C4A—C3A	112.23 (13)	N3C—C5C—H5C1	109.8
N3A—C4A—H4A1	109.2	C1C^iii^—C5C—H5C1	109.8
C3A—C4A—H4A1	109.2	N3C—C5C—H5C2	109.8
N3A—C4A—H4A2	109.2	C1C^iii^—C5C—H5C2	109.8
C3A—C4A—H4A2	109.2	H5C1—C5C—H5C2	108.3
H4A1—C4A—H4A2	107.9	N2D^iv^—Cr4D—N2D	180.0
N3A—C5A—C1A^i^	108.15 (12)	N2D^iv^—Cr4D—N3D	85.25 (5)
N3A—C5A—H5A1	110.1	N2D—Cr4D—N3D	94.75 (5)
C1A^i^—C5A—H5A1	110.1	N2D^iv^—Cr4D—N3D^iv^	94.76 (5)
N3A—C5A—H5A2	110.1	N2D—Cr4D—N3D^iv^	85.24 (5)
C1A^i^—C5A—H5A2	110.1	N3D—Cr4D—N3D^iv^	180.0
H5A1—C5A—H5A2	108.4	N2D^iv^—Cr4D—N1D^iv^	89.83 (5)
N2B^ii^—Cr2B—N2B	180.0	N2D—Cr4D—N1D^iv^	90.17 (5)
N2B^ii^—Cr2B—N3B	85.91 (6)	N3D—Cr4D—N1D^iv^	88.87 (6)
N2B—Cr2B—N3B	94.09 (6)	N3D^iv^—Cr4D—N1D^iv^	91.13 (6)
N2B^ii^—Cr2B—N3B^ii^	94.09 (6)	N2D^iv^—Cr4D—N1D	90.17 (5)
N2B—Cr2B—N3B^ii^	85.91 (6)	N2D—Cr4D—N1D	89.83 (5)
N3B—Cr2B—N3B^ii^	180.00 (7)	N3D—Cr4D—N1D	91.13 (6)
N2B^ii^—Cr2B—N1B	89.22 (6)	N3D^iv^—Cr4D—N1D	88.87 (6)
N2B—Cr2B—N1B	90.78 (6)	N1D^iv^—Cr4D—N1D	180.0
N3B—Cr2B—N1B	91.36 (5)	Cr4D—N1D—H1ND	109.5
N3B^ii^—Cr2B—N1B	88.64 (5)	Cr4D—N1D—H2ND	109.5
N2B^ii^—Cr2B—N1B^ii^	90.78 (6)	H1ND—N1D—H2ND	109.5
N2B—Cr2B—N1B^ii^	89.22 (6)	Cr4D—N1D—H3ND	109.5
N3B—Cr2B—N1B^ii^	88.64 (5)	H1ND—N1D—H3ND	109.5
N3B^ii^—Cr2B—N1B^ii^	91.36 (5)	H2ND—N1D—H3ND	109.5
N1B—Cr2B—N1B^ii^	180.00 (6)	C2D—N2D—C1D	114.07 (12)
Cr2B—N1B—H1NB	109.5	C2D—N2D—Cr4D	117.13 (10)
Cr2B—N1B—H2NB	109.5	C1D—N2D—Cr4D	107.05 (9)
H1NB—N1B—H2NB	109.5	C2D—N2D—H1D	105.9
Cr2B—N1B—H3NB	109.5	C1D—N2D—H1D	105.9
H1NB—N1B—H3NB	109.5	Cr4D—N2D—H1D	105.9
H2NB—N1B—H3NB	109.5	C4D—N3D—C5D	113.03 (12)
C2B—N2B—C1B	112.86 (13)	C4D—N3D—Cr4D	117.41 (10)
C2B—N2B—Cr2B	116.96 (11)	C5D—N3D—Cr4D	106.16 (9)
C1B—N2B—Cr2B	106.88 (10)	C4D—N3D—H2D	106.5
C2B—N2B—H1B	106.5	C5D—N3D—H2D	106.5
C1B—N2B—H1B	106.5	Cr4D—N3D—H2D	106.5
Cr2B—N2B—H1B	106.5	N2D—C1D—C5D^iv^	108.07 (12)
C4B—N3B—C5B	112.83 (13)	N2D—C1D—H1D1	110.1
C4B—N3B—Cr2B	117.59 (11)	C5D^iv^—C1D—H1D1	110.1
C5B—N3B—Cr2B	106.77 (10)	N2D—C1D—H1D2	110.1
C4B—N3B—H2B	106.3	C5D^iv^—C1D—H1D2	110.1
C5B—N3B—H2B	106.3	H1D1—C1D—H1D2	108.4
Cr2B—N3B—H2B	106.3	N2D—C2D—C3D	111.55 (13)
N2B—C1B—C5B^ii^	109.12 (14)	N2D—C2D—H2D1	109.3
N2B—C1B—H1B1	109.9	C3D—C2D—H2D1	109.3
C5B^ii^—C1B—H1B1	109.9	N2D—C2D—H2D2	109.3
N2B—C1B—H1B2	109.9	C3D—C2D—H2D2	109.3
C5B^ii^—C1B—H1B2	109.9	H2D1—C2D—H2D2	108.0
H1B1—C1B—H1B2	108.3	C4D—C3D—C2D	116.59 (14)
N2B—C2B—C3B	112.58 (14)	C4D—C3D—H3D1	108.1
N2B—C2B—H2B1	109.1	C2D—C3D—H3D1	108.1
C3B—C2B—H2B1	109.1	C4D—C3D—H3D2	108.1
N2B—C2B—H2B2	109.1	C2D—C3D—H3D2	108.1
C3B—C2B—H2B2	109.1	H3D1—C3D—H3D2	107.3
H2B1—C2B—H2B2	107.8	N3D—C4D—C3D	111.82 (13)
C2B—C3B—C4B	116.95 (15)	N3D—C4D—H4D1	109.3
C2B—C3B—H3B1	108.1	C3D—C4D—H4D1	109.3
C4B—C3B—H3B1	108.1	N3D—C4D—H4D2	109.3
C2B—C3B—H3B2	108.1	C3D—C4D—H4D2	109.3
C4B—C3B—H3B2	108.1	H4D1—C4D—H4D2	107.9
H3B1—C3B—H3B2	107.3	N3D—C5D—C1D^iv^	108.31 (12)
N3B—C4B—C3B	112.03 (13)	N3D—C5D—H5D1	110.0
N3B—C4B—H4B1	109.2	C1D^iv^—C5D—H5D1	110.0
C3B—C4B—H4B1	109.2	N3D—C5D—H5D2	110.0
N3B—C4B—H4B2	109.2	C1D^iv^—C5D—H5D2	110.0
C3B—C4B—H4B2	109.2	H5D1—C5D—H5D2	108.4
H4B1—C4B—H4B2	107.9	Cl4E—Zn1E—Cl1E	115.38 (3)
N3B—C5B—C1B^ii^	109.08 (13)	Cl4E—Zn1E—Cl3E	110.99 (3)
N3B—C5B—H5B1	109.9	Cl1E—Zn1E—Cl3E	108.75 (4)
C1B^ii^—C5B—H5B1	109.9	Cl4E—Zn1E—Cl2E	107.79 (3)
N3B—C5B—H5B2	109.9	Cl1E—Zn1E—Cl2E	107.77 (3)
C1B^ii^—C5B—H5B2	109.9	Cl3E—Zn1E—Cl2E	105.67 (3)
H5B1—C5B—H5B2	108.3	Cl1F—Zn2F—Cl4F	115.32 (3)
N3C^iii^—Cr3C—N3C	180.00 (6)	Cl1F—Zn2F—Cl2F	109.63 (3)
N3C^iii^—Cr3C—N2C^iii^	94.32 (6)	Cl4F—Zn2F—Cl2F	109.79 (4)
N3C—Cr3C—N2C^iii^	85.68 (6)	Cl1F—Zn2F—Cl3F	107.62 (3)
N3C^iii^—Cr3C—N2C	85.68 (6)	Cl4F—Zn2F—Cl3F	107.31 (2)
N3C—Cr3C—N2C	94.32 (6)	Cl2F—Zn2F—Cl3F	106.77 (4)
N2C^iii^—Cr3C—N2C	180.0	H1O1—O1W—H2O1	107.9 (19)
N3C^iii^—Cr3C—N1C	89.94 (6)	H1O2—O2W—H2O2	112.1 (19)
			
C2A—N2A—C1A—C5A^i^	171.57 (12)	C2C—N2C—C1C—C5C^iii^	170.49 (16)
Cr1A—N2A—C1A—C5A^i^	40.59 (14)	Cr3C—N2C—C1C—C5C^iii^	39.61 (18)
C1A—N2A—C2A—C3A	179.11 (12)	C1C—N2C—C2C—C3C	−177.36 (16)
Cr1A—N2A—C2A—C3A	−55.38 (15)	Cr3C—N2C—C2C—C3C	−52.33 (19)
N2A—C2A—C3A—C4A	70.45 (18)	N2C—C2C—C3C—C4C	67.1 (2)
C5A—N3A—C4A—C3A	178.01 (12)	C5C—N3C—C4C—C3C	−178.55 (14)
Cr1A—N3A—C4A—C3A	53.37 (15)	Cr3C—N3C—C4C—C3C	56.74 (17)
C2A—C3A—C4A—N3A	−69.39 (18)	C2C—C3C—C4C—N3C	−69.9 (2)
C4A—N3A—C5A—C1A^i^	−172.82 (12)	C4C—N3C—C5C—C1C^iii^	−167.98 (14)
Cr1A—N3A—C5A—C1A^i^	−42.24 (13)	Cr3C—N3C—C5C—C1C^iii^	−38.36 (16)
C2B—N2B—C1B—C5B^ii^	169.01 (14)	C2D—N2D—C1D—C5D^iv^	−171.39 (12)
Cr2B—N2B—C1B—C5B^ii^	39.06 (15)	Cr4D—N2D—C1D—C5D^iv^	−40.14 (13)
C1B—N2B—C2B—C3B	−179.75 (14)	C1D—N2D—C2D—C3D	−179.09 (13)
Cr2B—N2B—C2B—C3B	−55.15 (16)	Cr4D—N2D—C2D—C3D	54.77 (16)
N2B—C2B—C3B—C4B	68.20 (19)	N2D—C2D—C3D—C4D	−70.12 (19)
C5B—N3B—C4B—C3B	178.83 (14)	C5D—N3D—C4D—C3D	−177.52 (13)
Cr2B—N3B—C4B—C3B	53.86 (17)	Cr4D—N3D—C4D—C3D	−53.38 (16)
C2B—C3B—C4B—N3B	−67.16 (19)	C2D—C3D—C4D—N3D	69.25 (19)
C4B—N3B—C5B—C1B^ii^	−169.58 (14)	C4D—N3D—C5D—C1D^iv^	172.59 (12)
Cr2B—N3B—C5B—C1B^ii^	−38.92 (16)	Cr4D—N3D—C5D—C1D^iv^	42.50 (13)

Symmetry codes: (i) −*x*, −*y*+1, −*z*+2; (ii) −*x*+1, −*y*+1, −*z*+1; (iii) −*x*+1, −*y*+2, −*z*; (iv) −*x*, −*y*, −*z*+1.

### Hydrogen-bond geometry (Å, º)

**Table:**

*D*—H···*A*	*D*—H	H···*A*	*D*···*A*	*D*—H···*A*
N1*A*—H1*NA*···Cl3*F*^v^	0.90	2.80	3.3602 (16)	121
N1*A*—H1*NA*···Cl2*W*^vi^	0.90	2.60	3.3363 (18)	140
N1*A*—H3*NA*···Cl3*E*^vii^	0.90	2.58	3.384 (2)	149
N2*A*—H1*A*···Cl2*W*^vi^	0.99	2.20	3.1754 (15)	170
N3*A*—H2*A*···Cl3*F*^v^	0.99	2.30	3.2752 (14)	170
C1*A*—H1*A*2···Cl4*E*^vi^	0.98	2.59	3.4739 (19)	150
N1*B*—H2*NB*···Cl1*W*^ii^	0.90	2.45	3.2683 (19)	152
N1*B*—H2*NB*···O1*W*	0.90	2.46	3.034 (2)	122
N1*B*—H3*NB*···Cl2*E*^ii^	0.90	2.57	3.3556 (15)	147
N2*B*—H1*B*···Cl1*W*	0.99	2.20	3.1704 (17)	165
N3*B*—H2*B*···O1*W*^ii^	0.99	1.98	2.968 (2)	177
N1*C*—H2*NC*···Cl3*F*^viii^	0.90	2.68	3.4211 (19)	140
N1*C*—H3*NC*···Cl2*W*^ix^	0.90	2.38	3.2673 (17)	167
N1*C*—H3*NC*···O2*W*^ix^	0.90	2.54	2.975 (2)	111
N2*C*—H1*C*···O2*W*^x^	0.99	1.96	2.932 (2)	167
N3*C*—H2*C*···Cl2*W*^x^	0.99	2.23	3.2082 (16)	171
C5*C*—H5*C*2···Cl4*F*	0.98	2.79	3.761 (2)	174
N1*D*—H2*ND*···Cl1*W*^xi^	0.90	2.45	3.2796 (15)	154
N1*D*—H3*ND*···Cl1*E*^xi^	0.90	2.71	3.5516 (16)	156
N2*D*—H1*D*···Cl1*W*^xii^	0.99	2.19	3.1589 (16)	166
N3*D*—H2*D*···Cl2*E*^xii^	0.99	2.36	3.3276 (17)	166
C1*D*—H1*D*1···Cl1*F*^v^	0.98	2.66	3.6278 (17)	168
C1*D*—H1*D*2···Cl1*E*^xi^	0.98	2.83	3.803 (2)	172
O1*W*—H1*O*1···Cl1*W*^ii^	0.85 (1)	2.70 (2)	3.341 (2)	134 (2)
O1*W*—H2*O*1···Cl2*F*	0.84 (1)	2.39 (1)	3.2066 (17)	167 (2)
O2*W*—H1*O*2···Cl3*E*	0.83 (1)	2.37 (1)	3.1763 (19)	162 (2)
O2*W*—H2*O*2···Cl2*W*	0.84 (1)	2.55 (2)	3.2009 (19)	135 (2)

Symmetry codes: (ii) −*x*+1, −*y*+1, −*z*+1; (v) −*x*, −*y*+1, −*z*+1; (vi) *x*, *y*, *z*+1; (vii) *x*−1, *y*, *z*+1; (viii) *x*+1, *y*, *z*; (ix) −*x*+1, −*y*+1, −*z*; (x) *x*, *y*+1, *z*; (xi) −*x*+1, −*y*, −*z*+1; (xii) *x*−1, *y*, *z*.
